# Changes in crevicular cytokines after application of melatonin in patients with periodontal disease

**DOI:** 10.4317/jced.53934

**Published:** 2017-09-01

**Authors:** Javier Montero, Nansi López-Valverde, María-José Ferrera, Antonio López-Valverde

**Affiliations:** 1 Departament of Surgery, Faculty of Medicine, Scholl of dentistry, University of Salamanca, Spain; 2 Pinos Puente Health Centre, Granada-Metropolitan Health District, Granada, Spain

## Abstract

**Background:**

A clinical trial was designed to evaluate the effects of topical application of melatonin on the crevicular fluid levels of interleukins and prostaglandins and to evaluate changes in clinical parameters.

**Material and Methods:**

A consecutive sample of 90 patients were recruited from the Health Centre of Pinos Puente in Granada, Spain and divided into 3 groups: 30 patients with diabetes and periodontal disease, who were given melatonin; 30 patients with diabetes and periodontal disease, who were given a placebo, and 30 healthy individuals with no history of systemic disease or clinical signs of periodontal disease, who were also given a placebo. The 30 patients with diabetes and periodontitis were treated with topical application of melatonin (1% orabase cream formula) for 20 days by. The rest of the patients with diabetes and periodontitis and healthy subjects were treated with a placebo of orabase cream. We measured the gingival index by exploring the percentage of standing teeth bleeding on probing. The periodontogram was performed with a Florida Probe.

**Results:**

In the diabetic patients who were given topical melatonin, there was a statistically significant decrease in the two clinical parameters. By contrast, in diabetic patients who were given the topical placebo, there was no statistically significant variation.

**Conclusions:**

In patients with diabetes and periodontal disease, treatment with topical melatonin was associated with a significant improvement in the gingival index and in pocket depth, and a statistically significant reduction in concentrations of interleukin-1β, interleukin-6 and prostaglandin E2 in gingival crevicular fluid.

** Key words:**Melatonin, periodontal disease, diabetes mellitus, interleukin-1β, interleukin-6, prostaglandin E2.

## Introduction

Periodontal disease is an oral inflammatory process affecting the alveolar bone, the gums, and the periodontal ligament (1). In advanced periodontitis, there is extreme loss of gingival tissue and alveolar bone, with the possibility of tooth loss. The pathological mechanisms of periodontal disease are still not completely understood. Microbial organisms in dental plaque are considered the primary pathogens of periodontal disease (2), however the response of the host to the pathogens induces the production of inflammatory molecules, including cytokines and prostanoids, which are also involved in the initiation and progression of perio-dontal disease (3). Current knowledge of the pathogenesis of periodontitis suggests that it is a mixed infection in which the host response to bacterial biofilms is associated with high levels of pro-inflammatory mediators (3). These mediators trigger a cascade of events which, in some individuals, culminates in the irreversible degradation of connective and bone tissues, and consequent periodontal attachment loss (4). Certain individuals seem to be more susceptible to periodontal disease and the variability in the host response seems to be a major cause of disease extension and increased severity (5).

Many studies have shown that the biological activity of a variety of cytokines may be directly related to periodontal destruction (6). There is mounting evidence that cyclooxygenase plays an important role in the production of prostaglandin E2 (PGE2) in periodontal disease (6). Selective cyclooxygenase inhibitors are as effective as traditional non-steroidal anti-inflammatory drugs (NSAIDs) at inhibiting the progression of periodontal disease in animal models (7). In addition, interleukin-1β (IL-1β) and IL-6 are potent stimulators of prostaglandin production via cyclooxygenase-2 in human gingival fibroblasts (7). Il-6, IL-1β challenged human gingival fibroblasts induce a cyclooxygenase-2 expression via tyrosine kinase pathways (8). Numerous studies have shown elevated PGE2 levels in the gingiva and gingival crevicular fluid of patients with periodontal diseases compared to periodontally healthy subjects (9). Pro-inflammatory mediators, including Il-6, IL-1β and PGE2 are also associated with periodontal disease progression and alveolar bone resorption. Reductions in gingival crevicular fluid cytokines following initial periodontal therapy have also been reported (10).

Melatonin is an indoleamine secreted by the pineal gland in a circadian manner. It is now known that melatonin is produced in several organs and melatonin-forming enzymes are found in many tissues, including the retina, the ovaries, the gastrointestinal tract and immune system cells, among others (11). Melatonin has revealed itself to be a pleiotropic multitasking molecule (12). It is a noteworthy free radical scavenger and also plays an immunomodulatory role (12). In addition to powerful antioxidant activity, melatonin has anti-inflammatory effects, preventing over-expression of pro-inflammatory mediators and inhibiting the effects of several pro-inflammatory cytokines. The anti-inflammatory activity of melatonin has been consistently demonstrated in experimental and clinical studies (13,14) and it may be able to protect the oral cavity against free radicals produced by inflammatory diseases (15).

To further elucidate the anti-inflammatory activity of melatonin, we conducted clinical trials, aimed at determining the effect of topical application of melatonin on gingival crevicular fluid levels of IL-6, IL-1β and PGE2 in patients with diabetes and perio-dontal disease and evaluating the changes in two clinical parameters, gingival index and pocket depth, and the interrelation bet-ween them. We also aimed to analyse the association between the severity of the periodontal disease in diabetic patients and the increase in interleukin levels (IL-6, IL-1β and PGE2). Our hypothesis was that local application of melatonin improves the clini-cal parameters of the periodontal disease in diabetic patients with periodontitis and that there is a positive correlation between the levels of IL-6, IL-1β and PGE2 and the severity of the periodontal disease in patients with diabetes.

## Material and Methods

-Participants

We performed a consecutive selection of 90 patients from the Health Center in Pinos Puente (Granada, Spain) who met the inclusion/exlusion criteria over a period of 4 months. We used the following inclusion criteria: a) diagnosed with diabetes mellitus, type 1 or type II; b) currently receiving pharmacological treatment for diabetes (oral antidiabetics or insulin); c) levels of HbA1C ≥ 7%; and d) diagnosed with chronic periodontitis.

Patients had been previously diagnosed by their doctors according to the criteria established by the American Diabetes Association in 2010 (16): 1). Fasting plasma glucose ≥ 126 mg/dl (7.0 mmol/l), or 2). Glucose in plasma ≥ 200 mg/dl (11.1 mmol/l) at 2h during an oral glucose tolerance test or 3). Classic symptoms of hyperglycemia, or hyperglycemia crises, and the random plasma glucose test ≥200 mg/dl (11.1mmol/l), or 4). HbA1C ≥ 6.5%.

The clinical parameters of gingival index and pocket depth were used for the diagnosis of periodontitis.

Exclusion criteria were as follows: a) suffering from another important chronic inflammatory disease; b) the use of bisphosphonates, oral contraceptives, treatment with NSAIDs or antibiotics in the previous 6 months; c) being a smoker; d) being edentulous; e) not having the target teeth used in the Probe, and f) having received topical treatment for oral diseases, such as mouth-rinses or gels within the last 6 months.

We also selected from companions a group of healthy subjects with no evident oral or general pathologies of similar age and sex to those in the diabetic group, in order to compare the behaviour of crevicular fluid levels of IL-1β, IL-6 and PGE2 between healthy subjects and diabetic patients with periodontal disease.

A total of 90 people took part in the study. These were divided into 3 groups: Group 1 (DMPaMT): made up of 30 patients with diabetes and periodontal disease who were administered topical melatonin; Group 2 (DMPPlacebo): 30 patients with diabetes and periodontal disease, who were given a placebo, and Group 3 (NoDM): 30 healthy individuals with no history of systemic disease or clinical signs of periodontal disease, who were also given a placebo. The study was approved by the Ethics Committee of the Faculty of Odontology of the University of Granada (Spain). The research objectives were explained to the patients and later the written informed consent was obtained from all individual participants included in the study.

-Study Procedures

The study was performed in two phases. In phase 1, Group DMPaMT and DMPPlacebo were compared for the effects of topical application of melatonin or placebo, on gingival crevicular fluid levels of interleukin (IL)-1β, interleukin (IL)-6 and prostaglandin E2 (PGE2), and we assessed the changes in the clinical parameters (gingival index and pocket depth) in patients with diabetes and periodontal disease. In Phase 2 we compared the crevicular fluid levels of IL-1β, IL-6 and PGE2 for members of the group of diabetics with periodontitis with those for members of the control group (Group DMPaMT and NoDM).

All participants in the study underwent an oral examination, including medical, dental, and caries assessments. The same highly experienced dentist performed all the examinations. To determine the gingival index we follow the established criteria Löe & Silness, 1963 (17), directly using the bleeding and depth levels obtained by the Florida Probe, considered suitable for evaluating the changes before and after the application of the melatonin and the placebo. We examined the same teeth specified in the Community Periodontal Index (CPI). The periodontogram was performed using the Florida Probe handpiece (computerized periodontal probing system), taking three probing points per vestibular and three per palate and lingual, recording the probe depth. The scores of the probing depth are expressed in mm.

We then treated 30 patients with diabetes and periodontitis with topical application of melatonin (1% orabase cream formula) in both the upper and lower dental arches on the surfaces of the attached gingiva for 20 days. The other patients with diabetes and periodontitis and the healthy subjects were treated with a placebo orabase cream. Melatonin and placebo were assigned to diabetic periodontal patients at random by using blank closed envelopes. All the participants were instructed as to how to use the melatonin cream (or placebo), which they applied daily at night after routine oral hygiene; for each dental arch they were recommended to apply the amount that fits on a normal adult toothbrush. They used their toothbrushes as a reference for the amount of cream to use, spreading the gel across their gums. They were instructed not to brush this area, just to apply the cream. In the morning they went through their normal dental hygiene routine. All the participants were given the same brand of toothpaste for use during the course of the study. Conventional periodontal treatment prior to or during the study was not allowed. All the baseline examinations were also performed just at the end of the treatment (20 days after). The examiner was blinded regarding the type of ointment (melatonin/placebo).

Melatonin was purchased in pure state from a company called Helsinn Advanced Synthesis SA. The melatonin in orobase gel for oral use at 1% was made by the Perpetuo Socorro Company (from Granada, Spain), who also provided the gel with the placebo. The excipient for the placebo had identical characteristics to the gel with the melatonin. The quality certificate was provided by: Metapharmaceutical IND, SL. Jose Pla 163-Barcelona-Spain.

-Measurements of Gingival Crevicular Fluid IL-1β, IL-6 and PGE2

Periodontal pockets were sampled for gingival crevicular fluid, which was collected using paper strips (Periopaper strip, Harco Medical Electronic Devices, Tustin, CA, USA) prior to any periodontal probing. Samples were collected at two sites (mesio-buccal and disto-buccal) from posterior teeth in each quadrant, excluding the third molars (9,18). The site was isolated and air-dried, and the fluid volume in each strip was measured by a calibrated electronic gingival fluid measuring device (18). IL-1β, IL-6 and PGE2 concentrations in the fluid were measured separately using ELISA (Cayman Chemical, Ann Arbor, MI, ISA). Results were expressed as ng/mL (IL-1β and PGE2) and as pg/ml (IL-6).

-Statistical Analysis

The Shapiro-Wilk test was applied to verify the normal distribution of the continuous variables. Continuous variables are expressed as the mean ± standard deviation (SD) if normally distributed. The Chi-squared test was used for the categorical variables. The paired Student’s t test was used for the comparison of the gingival index and the probing depth before and after topical application of melatonin among diabetic patients with periodontal disease, and the ANOVA and Student’s t test for independent samples was used for comparing the crevicular fluid levels of IL-1β, IL-6 and PGE2 of the groups of diabetic patients and of healthy subjects. The relationship between the gingival index and the probing depth with crevicular fluid IL-1β, IL-6 and PGE2 was assessed with the Pearson’s correlation coefficient. Statistical significance was set at *p* < 0.05. The Statistical analyses were performed using the R-Commander (version 2.15.1 (2012-06-22).

## Results

The sample groups were shown to be homogenous in terms of age and sex, being mostly adults between 40-50 yrs and slightly higher proportion of females ( Table 1).

**Table 1:**
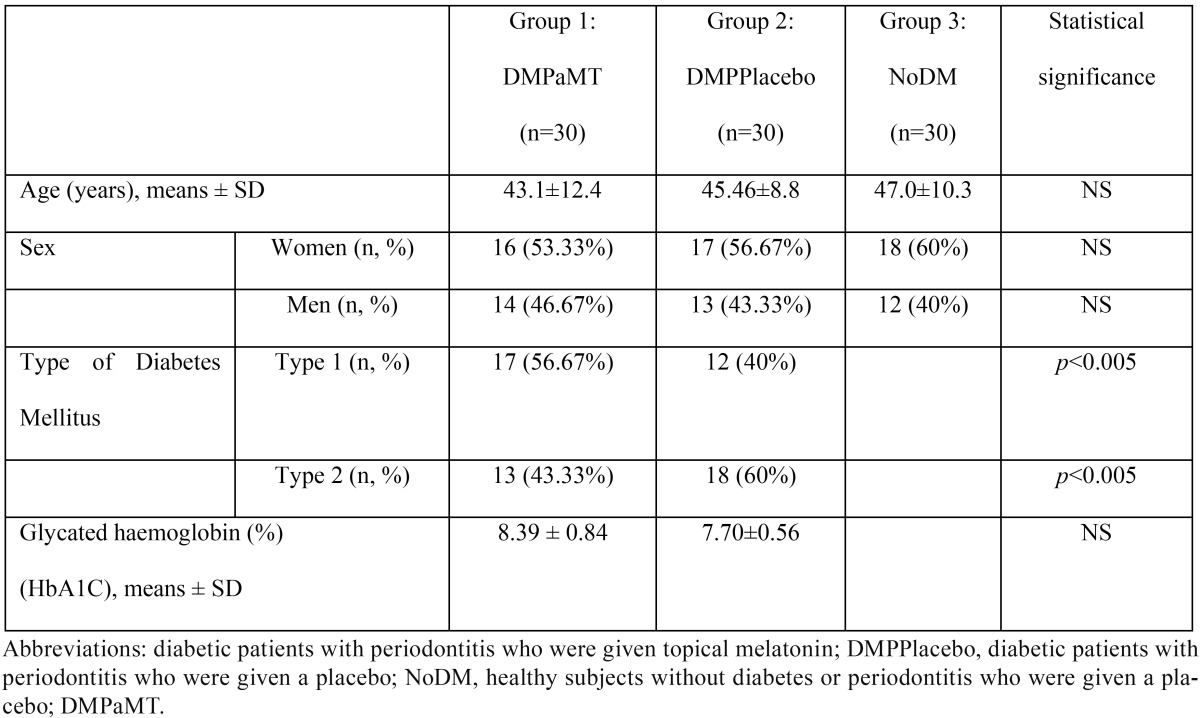
Sociodemographic and clinical parameters of the three group of subjects.

The sociodemographic and clinical characteristics of the patients by groups were: Group 1 (DMPaMT): 30 patients with diabetes and periodontal disease (53.33% women) aged between 24 and 58 years (43.1 ± 12.4 yrs on average), 17 patients with type I diabetes (56.67%) and 13 with type II diabetes (43.33%); The Group 2 (DMPPlacebo): 30 patients with diabetes and periodontal disease (56.7% women) aged between 29 and 59 years (45.46 ± 8.8 yrs on average), 12 patients with type I diabetes (40%) and 18 with type II diabetes (60%). Group 3 (NoDM): 30 healthy individuals (60% women), aged between 31 and 68 years (47.0 ± 10.3 yrs on average) without periodontal disease. The baseline Glycated haemoglobin levels (HbA1C %) in the diabetic groups were 8.4% ± 0.5 for the group that received topical melatonin and 7.7%±0.56 for the group that received the placebo. This difference was not significant ( Table 1).

The comparison of gingival crevicular fluid levels of IL-1β, IL-6 and PGE2 between the different groups of diabetic patients with periodontal disease and healthy controls before topical treatment with melatonin or the placebo are shown in  Table 2. Patients with diabetes and periodontal disease from group DMPaMT and group DMPPlacebo had significantly higher mean levels of IL-1β (127.73±99.50 and 122.47±95.2 ng/mL), IL-6 (0.57±0.56 and 0.56±0.007 pg/mL) and PGE2 (265.42±101.6 and 263.45±98.7 ng/mL) than healthy subjects (IL-1β (95.35±59.26 ng/mL), IL-6 (0.38±0.005 pg/mL) and PGE2 (205.71±118.09 ng/mL). However there were no significant differences between the two groups of diabetic patients ( Table 2).

**Table 2:**
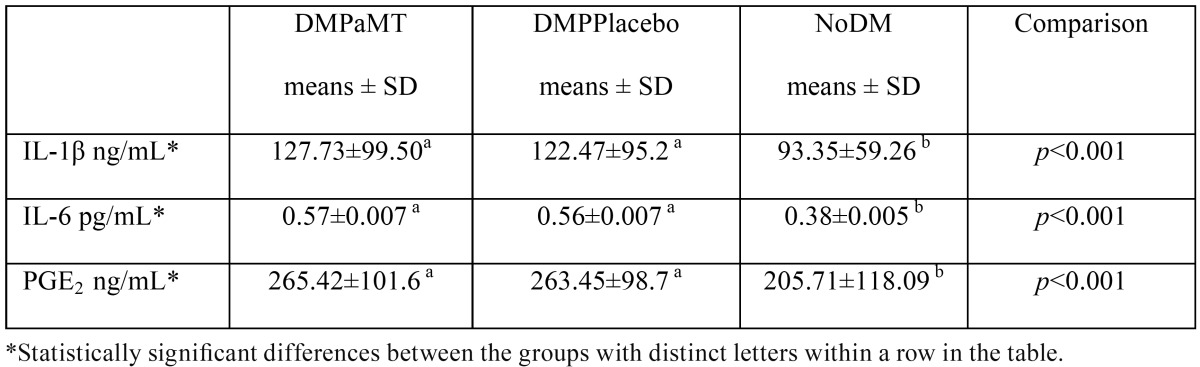
Gingival crevicular fluid levels of IL-1β, IL-6 and PGE2 in patients from the three study groups before topical treatment.

In the diabetic patients that received topical melatonin, there was a statistically significant decrease (before/after) in the gingival index (15.84 ± 10.26 vs 5.59 ± 4.08) and pocket depth (2.8 ± 1.9 vs 1.8 ± 1.2) as well as a significant decrease in gingival crevicular fluid levels of IL-1β (127.73 ± 99.50 vs 114.34 ± 74.88 ng/mL, *P* = 0.012), IL-6 (0.57 ± 0.07 vs 0.47 ± 0.07 pg/mL, *P* <0.001) and PGE2 (265.42 ± 101.60 vs 222.78 ± 87.88 ng/mL, *P* < 0.001) ( Table 3). However in the diabetic patients who received the topical placebo, there was no statistically significant change (before/after) in the gingival index (14.51 ± 9.70 vs 14.13 ± 10.15) and the pocket depth (2.7 ± 1.5 vs 2.6 ± 1.2), although there was a mild decrease in gingival crevicular fluid levels of IL-1β (122.47 ± 95.2 vs 120.93 ± 101.4 ng/mL), IL-6 (0.56 ± 0.07 vs 0.54 ± 0.07 pg/mL) and PGE2 (263.45 ± 98.7 vs 260.26 ± 99.1 ng/mL) as it is shown in  Table 3.

**Table 3:**
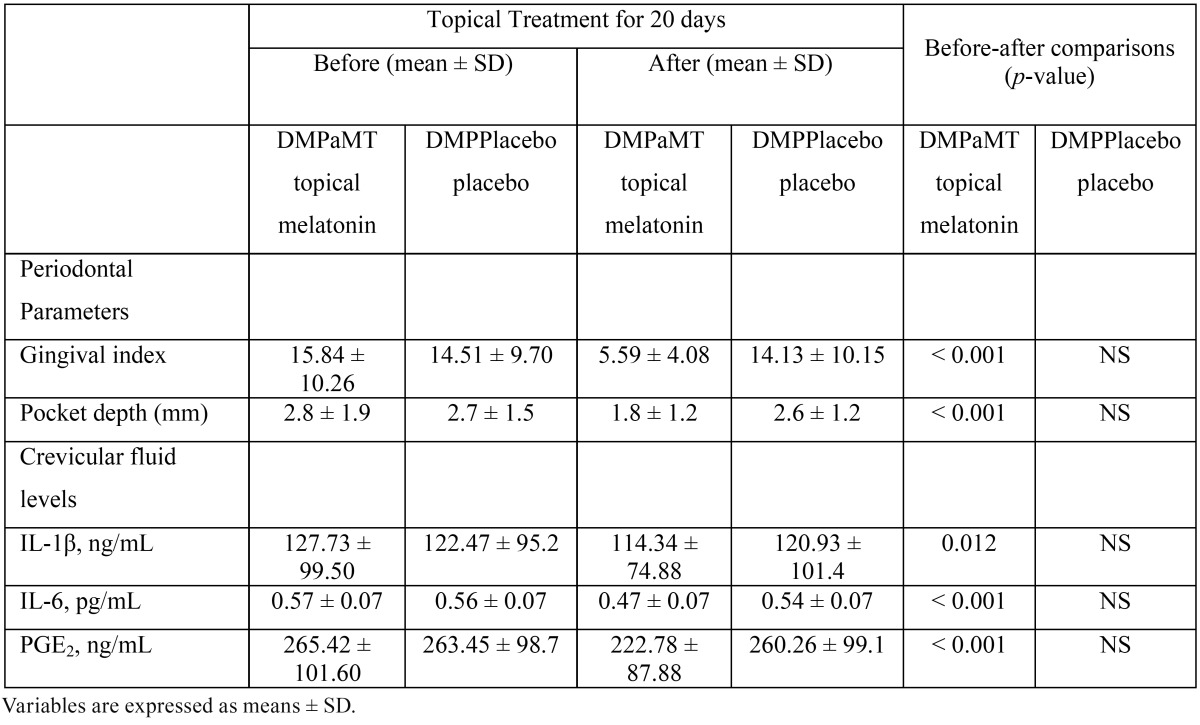
Comparison of gingival index, pocket depth and gingival crevicular fluid levels of IL-1β, IL-6 and PGE2 before and after topical application of melatonin or placebo in diabetic patients with periodontal disease (DMPaMT and DMPPlacebo).

There was also a direct and significant correlation between the gingival crevicular fluid levels of IL-1β, IL-6 and PGE2 and the periodontal parameters (gingival index and pocket depth) both at baseline and after the topical melatonin treatment ( Table 4). These correlation coefficients were higher for the IL-1β and PGE2 than for the IL-6, and were stronger for the baseline parameters than after treatment.

**Table 4:**
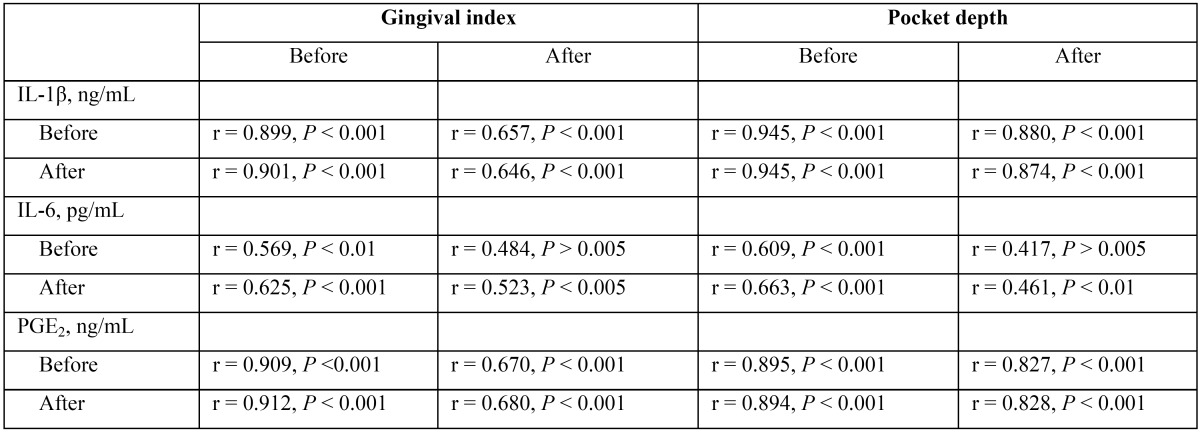
Correlation between the periodontal parameters (gingival index and pocket depth) with the gingival crevicular fluid levels of IL-1β, IL-6 and PGE2 before and after treatment with melatonin in diabetic patients with periodontal disease. (DMPaMT).

## Discussion

These results confirm the beneficial effects of the topical application of melatonin on clinical parameters for periodontal disease, such as the gingival index and the pocket depth. All comparisons before and after treatment with melatonin were statistically significant. Moreover, the anti-inflammatory action of melatonin was demonstrated by a significant decrease in cytokines IL-1β, IL-6 and PGE2 in gingival crevicular fluid of patients with diabetes and periodontal disease (19). However, to assure that these treatment outcomes are because of the topical use rather than a systemic effect we should have analysed blood samples for quantifying melatonin levels before and after the topical treatment. Because since the melatonin was applied to the gingiva over 20 days on a daily basis, it may be plausible that part of the ointment was swallowed and worked systemically.

Before treatment with melatonin, levels of IL-1β, IL-6 and PGE2 in gingival crevicular fluid of patients with diabetes and perio-dontal disease were significantly higher than in healthy control subjects without periodontitis. We should not forget that human adipose tissue is a potent source of inflammatory interleukins that could potentially lead us to a mistaken conclusion regarding a possible relationship between periodontal illness and the presence of interleukins (18), because in this study we did not measure the patient’s obesity (Body Mass Index. Our findings confirm that treatment with topical melatonin of the periodontal disease among diabetic patients reduce significantly the presence of crevicular interleukins. Furthermore previous studies have shown higher IL-1β and PGE2 crevicular fluid levels in subjects with either type 1 or type 2 diabetes than in subjects without diabetes, regardless of periodontal status (9,20). Probing depth and gingival index have been reported to be significantly higher in poorly controlled diabetes compared to systemically healthy subjects, with positive associations between periodontal inflammation and levels of IL-1β in the gingiva and in gingival crevicular fluid (21,22). It has been suggested that hyperglycemia may induce inflammatory cytokine production (23). These studies suggest a possible dysregulation of the normal cytokine/growth factor signalling axis in poorly controlled diabetes that may contribute to periodontal breakdown/diminished repair (9). Overexpression of IL-6 and IL-1β in periodontally inflamed tissue has also been postulated as a mechanism by which type 2 diabetes enhances periodontal destruction (24).

IL-1β is a potent bone-resorptive cytokine that also mediates soft-tissue destruction by stimulating prostaglandin production and inducing collagenase and other protease activity. The literature suggests that this substance may be an important mediator of attachment loss in human periodontitis, and indicates that IL-1β may be useful for locating sites of periodontal disease activity (20). Studies on the determination of the IL-1 signaling cascades that lead to the production of various inflammatory mediators have shown that MAPK, AP-1 and NF-κB mediate the IL-1β-stimulated synthesis of IL-6, IL-8, PGE2 and MMP-1 in human periodontal ligament cells (25). Therefore, inhibition of activation of MAPK, AP-1 and/or NF-κB may lead to therapeutic effects in terms of the progression of periodontitis (25). In addition, periodontal ligament cells secrete IL-10, which can be suppressed by IL-1β and it has been hypothesized that periodontal ligament cells can function as accessory immunoinflammatory cells amplifying the inflammatory process in periodontitis, thereby contributing to periodontal breakdown (26). Periodontal ligament cells activated by inflammatory factors such as IL-1β and PGE2 may also directly stimulate osteoclastogenesis through RANKL (27).

For its part, IL-6 is an important pro-inflammatory cytokine involved in the regulation of host response to tissue injury and infection. It is produced by a variety of cells, such as monocytes, fibroblasts, osteoblasts and vascular endothelial cells in response to inflammatory challenges (27). In our study, as in others (28), high levels of IL-6 were found in patients with periodontal disease as compared with healthy controls.

Periodontal therapy has been shown to reduce levels of IL-1β in the gingival crevicular fluid. Removal of the bacterial plaque reduces the antigenic stimuli and consequently could modulate the chemokines present in the gingival crevicular fluid (29). In general our results coincide with the findings of these authors, although in our study periodontal treatment was replaced by topical application of melatonin, whose positive effects in ameliorating periodontal disease had been demonstrated previously (15).

## Conclusions

Treatment of patients with diabetes and periodontal disease with topical melatonin was associated with a significant improvement in the gingival index and in pocket depth, and a statistically significant reduction in concentrations of IL-1β, IL-6 and PGE2 in gingival crevicular fluid.

Patients with diabetes and periodontal disease had significantly higher mean levels of IL-1β, IL-6 and PGE2 than healthy subjects.

There was a direct and strong correlation between clinical periodontal parameters and the changes in crevicular fluid levels of IL-1β, IL-6 and PGE2 before and after topical melatonin treatment.
